# A STORM IS COMING: A CLIMATE RESILIENCE FRAMEWORK FOR SENIOR HOUSING

**DOI:** 10.1093/geroni/igae098.1378

**Published:** 2024-12-31

**Authors:** Taylor Patskanick, Grace Turner, Joseph Coughlin

**Affiliations:** Massachusetts Institute of Technology, Cambridge, Massachusetts, United States; Massachusetts Institute of Technology, Cambridge, Massachusetts, United States; MIT AgeLab, Cambridge, Massachusetts, United States

## Abstract

Older adults’ vulnerability to extreme weather events may vary especially based on geographic location and housing or residential situation. Emergency preparedness is a potentially modifiable protective factor influencing this vulnerability. Emergency preparedness, however, looks different for older adults living in senior housing communities compared to community-dwelling older adults. For example, the responsibility for emergency preparedness for extreme weather within the senior housing environment expands to include the housing operator and community-based emergency management services rather than this responsibility relying solely on the individual older adult. This paper examines a set of twenty interviews conducted with senior housing operators, owners, and community-based emergency services personnel located in five U.S. states exploring emergency preparedness for extreme weather events in senior housing. Interviews were analyzed using an inductive, team-based thematic analysis approach. Results from this study are presented in a three-phase modified Haddon Matrix (Haddon, 1968) to provide a comprehensive framework promoting age-friendly extreme weather preparedness for senior housing operators and local emergency services. The implications of this research offers a foundation for a standardized checklist of actions and processes for senior housing operators, their individual communities, and emergency services to use to build capacity for age-friendly climate resilience. Senior housing communities serving older adults must comprehensive and resilient emergency preparedness plans and programs able to respond to a changing climate in an ongoing, adaptive, and multi-phase way that accounts for all actors in the senior housing environment (e.g., staff, residents, resident families) and through every stage of an extreme weather event.

